# Our Own

**Published:** 1875-11

**Authors:** 


OUR OWN.

If I had known in the morning
How wearily all the day
The words unkind
Would trouble my mind
I said when you went away,
I had been more careful, darling,
Nor given you needless pain;
But we vex "our own"
With look and tone
We may never take back again.

For though in the quiet evening
You may give us the kiss of peace,
Yet it might be
That never for me
The pain of the heart should cease.
How many go forth in the morning
That never come home at night!
And hearts have broken
For harsh words spoken
That sorrow can ne'er set right.

We have careful thoughts for the stranger
And smiles for the sometimes guest;
But oft for "our own"
The bitter tone,
Though we love our own the best.
Ah! lips with the curve impatient!
Ah! brow with the look of scorn!
'Twere a cruel fate
Were the night too late
To undo the work of morn.

THINGS are pretty well balanced in this world, so far as taking comfort goes, and I begin to believe that high or low, all have their tribulations. Fishes are hooked, worms are trodden on, birds are fired at. Worry is everywhere. Poor men's wives worry because the bread won't rise, or the stove won't draw, or the clothes line breaks, or the milk burns, or the pane of glass is mended with putty, or they can't afford to hire help. Rich men's wives worry because the preserve dish is not of the latest pattern, or because somebody finds out how a party dress is trimmed before the party happens; or because some grandee's wife overlooks them, or because their help 'sauces' them, breaks up tea sets, spoils dinners, gets drunk, and cuts up sheets into underclothes. Causes vary, but worry averages about the same. The scale of miles is different on different maps, but places remain just so far apart, and so do humanity and content.

---

Never put off until to-morrow what you can do to-day. Never trouble another for what you can do yourself. Never spend your money before you have it. Never buy what you don't want because it is cheap. Pride costs more than hunger, thirst, and cold. We seldom repent of having eaten too little. Nothing is troublesome that we do willingly. How much pain the evils have cost us that never happened! Take things always by the smooth handle. When angry count ten before you speak; if very angry count a hundred.

